# Effect of family management styles on the outcomes of children with complex congenital heart disease after palliative surgery

**DOI:** 10.3389/fped.2025.1555982

**Published:** 2025-03-27

**Authors:** Yuxian Xia, Rui Yang, Yuemeng Zhang, Di Yin, Wen Zhang, Qi Jiang, Yifan Zhu, Haibo Zhang, Renjie Hu, Wei Dong

**Affiliations:** ^1^ Department of Cardiothoracic Surgery, Shanghai Children’s Medical Center, Shanghai Jiaotong University School of Medicine, Shanghai, China; ^2^ Shanghai Jiaotong University School of Medicine, Shanghai, China

**Keywords:** congenital heart disease, palliative surgery, family management styles, pediatric quality of life, surgical outcome

## Abstract

**Background:**

This study aimed to explore family management style (FMS) after palliative surgery in children with complex congenital heart disease (CCHD) and evaluate its influence on their outcomes.

**Methods:**

A cross-sectional survey was conducted among 252 families of children with CCHD who underwent palliative surgery at our center. The Family Management Measure was used to investigate their FMS, and the outcomes with different FMSs were analyzed. Cluster analysis was employed to classify the FMSs into distinct groups.

**Results:**

The cluster analysis identified four FMSs, namely, the Active and Collaborative (Cluster 1, 29.37%), the Chaotic and Nervous (Cluster 2, 10.71%), the Confident and Caring (Cluster 3, 22.22%), and the *Laissez-Faire* style (Cluster 4, 37.70%). Children in Cluster 1 demonstrated the highest quality of life, while those in Cluster 2 had the lowest (73.93 ± 12.71 and 59.03 ± 18.70, *P* < 0.01). The unplanned readmission rates were significantly higher in Clusters 2 and 4 (18.52% and 22.11%) compared to Clusters 1 and 3 (4.05% and 3.57%, *P* < 0.01).

**Conclusion:**

The findings highlight the significant influence of FMS on the outcomes of children with CCHD following palliative surgery. The children in Cluster 1 exhibited the most favorable quality of life, whereas those in Cluster 2 had the worst. Health professionals should implement interventions to optimize FMS.

## Introduction

Congenital heart disease (CHD) is the most prevalent group of congenital anomalies worldwide, diagnosed in approximately 1% of live births (1). China is no exception, as CHD is the leading cause of neonatal impairment and mortality in the country. Infants with complex defects often require multiple stages of palliative and corrective surgeries early in life, followed by extended hospitalizations in the cardiac intensive care unit (CICU). Children born with complex congenital heart disease (CCHD) are particularly medically fragile and require intensive medical monitoring after discharge. They frequently experience feeding difficulties and growth delays and remain at risk for congestive heart failure (2).

The families of children with CCHD must adapt to postoperative caregiving and manage their children's condition in the long term, as their children often require palliative surgeries to alleviate clinical symptoms and prolong life. Their complex condition cannot be fully corrected anatomically or relieved in a single visit (3). Compared to radical surgery for simple CHD, children undergoing palliative procedures are more prone to unexpected complications due to hemodynamic abnormalities caused by non-anatomical correction (4).

An increasing body of literature indicates that postoperative care for children following palliative surgery for CCHD is suboptimal. Delayed growth and unplanned readmission rates are increasing (5). In addition, mortality rates can reach up to 10%–15% after certain palliative surgeries (4). Moreover, postoperative care for children with CCHD remains a challenging process for most parents, as these families often face significant ongoing pressure in China (6). Therefore, it is essential for healthcare professionals to support these families in developing comprehensive care plans and to help them maintain an active role in the long-term care of their children.

The Family Management Style Framework (FMSF) identifies how families organize, integrate, and carry out family-related tasks when managing chronic health conditions in children (7). A qualitative study explored the potential practical and research implications of the FMSF for children with CHD and demonstrated how family management style (FMS) dynamically influences children's health outcomes over time (8). In addition, their findings suggested that higher survival rates were associated with parents who exhibited a thriving FMS, held more positive attitudes toward their children's condition, and were more confident in managing their children's care regimen (8). Therefore, we aimed to conduct a large-scale quantitative study to quantify the impact of FMS on the outcomes of children with CCHD who underwent palliative surgeries. This study sought to extend our understanding of the application of FMS in this population and provide concrete evidence to inform postoperative care strategies for these children.

## Methods

### Study population

The families of children with CCHD who underwent palliative surgeries at Shanghai Children's Medical Center from 1 January 2016 to 31 August 2021 and who signed the consent form to take part in this study were recruited.

Inclusion criteria: (1) the palliative surgeries must have been performed within the last 5 years; (2) the caregivers must be the child's parents; (3) the parents must be able to communicate accurately and freely; (4) the parents must have agreed to participate in the study.

Exclusion criteria: (1) children born prematurely; (2) children with chromosomal abnormalities; (3) children with serious postoperative complications requiring long-term medical intervention.

### Data collection and assessments

The general information was collected through surveys. Diagnoses were classified as either single ventricular malformation or biventricular malformation. In addition, data on the children's family location, family economic status, and parents’ education and occupation were collected.

Cardiac function assessment during follow-up was performed on all children using the New York Heart Association (NYHA) cardiac function grade (9), which is divided into grades I–IV.

A nutritional status assessment was conducted using weight-for-age *Z*-score (WAZ) and body mass index (BMI)-for-age. WAZ was used to evaluate the nutritional status of children younger than 2 years old, with a score of less than -2 indicating a risk of malnutrition. BMI-for-age was utilized to assess the nutritional status of children older than 2 years old, with a BMI percentile less than 5% indicating malnutrition.

The Family Management Measure (FaMM) was initially developed by Knafl in 2006 based on the FMSF (7). The Chinese version of the FaMM was translated by Ying Zhang and her colleagues using the Likert 5-point scale, demonstrating good reliability and validity (10). The scale consists of 53 items across six dimensions. These include three positive dimensions: the Children's Daily Life (CDL) subscale, Condition Management Ability (CMA) subscale, and Parental Mutuality (PM) subscale. Higher scores in these dimensions indicate better family management. In addition, there are three negative dimensions: the View of Condition Impact (VCI) subscale, Family Life Difficulty (FLD) subscale, and Condition Management Effort (CME) subscale. Higher scores in these dimensions indicate poorer family management. The Cronbach’*α* for each subscale ranges from 0.70 to 0.84, and the content validity index (CVI) is 0.84 (10, 11).

Pediatric Quality of Life (PedsQL^MT^) is a measurement scale developed by Professor Upton to quantify children's quality of life (12), including two parts, the Child Self-Assessment Questionnaire and the Caregiver Report Questionnaire. This study used a Chinese version of the universal core PedsQL^MT^4.0 Caregiver Report Questionnaire, which has a reliability ranging from 0.74 to 0.90 (13). The questionnaire includes 23 items across four dimensions: physical functioning, emotional functioning, social functioning, and school functioning. Each item requires participants to recall how often an event occurred in the previous month, and responses are scored using a 5-point Likert scale. A higher score indicates a better quality of life (13).

The socioeconomic status (SES) index was used to measure parents’ occupational prestige and SES. The calculation formula is as follows: SES Index = 11.808 + 3.349 × (average years of education) + 0.573 × (average monthly income in 100 yuan) + 16.075 × (maximum managers) + 11.262 × (middle managers) + 3.738 × (grassroots managers) + 8.942 × (Party and government agencies) + 6.841 × (institutions) − 5.694 × (business units) − 26.655 × (discriminated occupations).

### Statistical analysis

Descriptive statistics were reported as mean (standard deviation) or median [interquartile range (IQR)] for continuous variables and as percentages or frequencies for categorical variables.

A cluster analysis method was employed to identify different family management styles based on the FaMM dimension scores involving three steps. First, a cluster analysis, involving systematic clustering that determined the number of possible clusters according to the pedigree and inter-group connection. Second, K-means cluster, an iterative partitioning method, was performed to identify the best clustering results so that each cluster must contain at least 5% of the entire sample. Third, one-way analysis of variance (ANOVA) and a *post-hoc* test (Bonferroni test) were then used to test the differences in subscales between each cluster.

Differences in continuous or categorical variables across family management styles were assessed by ANOVA or chi-square tests, and the corresponding *X*^2^, F, and *P*-values are reported in the tables. A *P*-value <0.05 (two-tailed) was considered statistically significant. All statistical analyses were performed using SPSS 19.0.

## Results

### Patient characteristics

A total of 269 families of children with CCHD within 5 years after palliative surgery were followed up in this study, and 252 families completed the questionnaire. The effective response rate was 93.68%, indicating that the results were representative of the target population. The median age of the children at surgery was 2.44 years (IQR: 0.71–6.99 years), the median follow-up age was 4.18 years (IQR: 2.38–8.71 years), the median CICU stay duration was 3.81 days (IQR: 2.00–6.72 days), and the median ventilation time was 26.68 h (IQR: 8.51–79.27 h).

### Cluster analysis

The cluster analysis identified common characteristics within groups by measuring a predefined set of variables (the mean scores of each FaMM subscale). Consequently, the label assigned to each group reflected its typical characteristics. The clustering process was guided by the following principles: appropriate sample size, best interpretability within each cluster, *F*-values, and one-way ANOVA results. Based on these criteria, the included families were divided into four distinct family management styles (four clusters).

These four clusters were characterized as Active and Collaborative (Cluster 1), Chaotic and Nervous (Cluster 2), Confident and Caring (Cluster 3), and *Laissez-Faire* style (Cluster 4). Detailed descriptions of these clusters are provided in Table 1.

**Table 1:** Mean scores of six FaMM dimensions in the four clusters (mean ± SD).

FaMM	Cluster 1 (*n* = 74)	Rank	Cluster 2 (*n* = 27)	Rank	Cluster 3 (*n* = 56)	Rank	Cluster 4 (*n* = 95)	Rank	Eta^2^	*F*	*P*
CDL	20.62 ± 2.23	1^a^	13.04 ± 3.35	4^d^	18.64 ± 3.36	2^b^	15.07 ± 2.42	3^c^	0.51	85.07	<0.001
CMA	44.88 ± 4.28	2^a^	36.07 ± 5.11	4^c^	45.66 ± 5.40	1^a^	40.00 ± 4.32	3^b^	0.33	41.11	<0.001
PM	32.70 ± 4.40	1^a^	13.67 ± 13.02	4^d^	30.05 ± 4.19	2^b^	26.95 ± 6.38	3^c^	0.42	58.63	<0.001
VCI	27.57 ± 3.78	4^c^	36.70 ± 5.29	2^a^	38.96 ± 4.94	1^a^	31.43 ± 4.20	3^b^	0.50	81.78	<0.001
FLD	32.39 ± 6.15	4^c^	53.41 ± 7.91	1^a^	50.82 ± 7.35	2^a^	40.21 ± 5.90	3^b^	0.59	116.49	<0.001
CME	12.05 ± 2.71	3^b^	16.41 ± 3.02	1^a^	15.18 ± 2.57	2^a^	11.96 ± 2.79	4^b^	0.28	32.77	<0.001

FaMM, Family Management Measure; SD, standard deviation; CDL, Children's Daily Life subscale; CMA, Condition Management Ability subscale; PM, Parental Mutuality subscale; VCI, View of Condition Impact subscale; FLD, Family Life Difficulty subscale; CME, Condition Management Effort subscale.

Values were ranked from the highest to the lowest.

Differences in the *post-hoc* test are represented in the following order: a > b > c > d, *P* < 0.05.

#### Cluster 1: Active and Collaborative style

This cluster accounted for 29.37% (*n* = 74) of the sample. In this cluster, the CDL and PM subscales had the highest scores, while the two negative dimensions, VCI and FLD, had the lowest scores. The parents in this cluster demonstrated a natural acceptance of their children's condition. Despite their children having CCHD requiring palliative surgery, they were able to effectively manage both the children and the disease across various aspects of family life.

#### Cluster 2: Chaotic and Nervous style

This cluster accounted for 10.71% (*n* = 27). In this cluster, the scores for FLD and CME were the highest, while the scores for the three positive dimensions (CDL, CMA, and PM) were the lowest. The parents in this cluster struggled to address their children's conditions calmly and exhibited excessive worry. Most of the parents in this cluster lacked confidence in overcoming challenges and managing their children's conditions, leading to significant consumption of energy and time. In addition, mutual support between the parents was notably lower compared to the other three clusters. Many of the parents in this cluster experienced dissatisfaction or conflict and demonstrated neither the ability nor the intention to understand the medical issues related to their children.

#### Cluster 3: Confident and Caring style

This cluster accounted for 22.22% (*n* = 56). In this cluster, CMA and VCI scored the highest. The parents in this cluster were capable of caring for their children but expressed concerns about the disease's impact on their children and families.

#### Cluster 4: *Laissez-Faire* style

This cluster accounted for 37.70% (*n* = 95). In this cluster, the score of CME ranked lowest, while VCI and FLD were also low. Parents in this cluster invested the least amount of energy, and the scores for the three positive dimensions were significantly lower than those in Clusters 1 and 3 (*P* < 0.05).

### Characteristics of children and families with different family management styles

There were no significant differences in gender, nutritional status, times of surgery, family location, and paternal SES index distribution among the four clusters (*P* > 0.05). However, significant differences were observed in the distributions of children's age, cardiac function grade, diagnosis, time from surgery to follow-up, and maternal SES index (all *P* < 0.05) across the four clusters (Table 2).

**Table 2:** The characteristics of the children and families in the four clusters.

Characteristics	Cluster 1, *n* (%)	Cluster 2, *n* (%)	Cluster 3, *n* (%)	Cluster 4, *n* (%)	Total, *n* (%)	*X* ^2^ */F*	*P*
Gender						5.31	0.153
Boy	51 (68.92)	19 (70.37)	30 (53.57)	53 (55.79)	153 (60.71)		
Girl	23 (31.08)	8 (29.63)	26 (46.43)	42 (44.21)	99 (39.29)		
Cardiac function						18.04	0.035
Grade I	14 (18.92)	6 (22.22)	15 (26.79)	27 (28.42)	62 (24.60)		
Grade II	36 (48.65)	6 (22.22)	21 (37.50)	33 (34.74)	96 (38.10)		
Grade III	20 (27.03)	8 (29.63)	18 (32.14)	24 (25.26)	70 (27.78)		
Grade IV	4 (5.41)	7 (25.93)	2 (3.57)	11 (11.58)	24 (9.52)		
Nutritional status						0.82	0.845
Normal	39 (52.70)	12 (44.44)	27 (48.21)	50 (52.63)	128 (50.79)		
Malnutrition	35 (47.30)	15 (55.56)	29 (51.79)	45 (47.37)	124 (49.21)		
Diagnosis						12.86	0.005
Single ventricular malformation	35 (47.30)	12 (44.44)	10 (17.86)	36 (37.89)	93 (36.90)		
Biventricular malformation	39 (52.70)	15 (55.56)	46 (82.14)	59 (62.11)	159 (63.10)		
Number of surgeries						7.68	0.265
One	24 (32.43)	7 (25.93)	13 (23.21)	35 (36.84)	79 (31.35)		
Two	32 (43.24)	14 (51.85)	31 (55.36)	49 (51.58)	126 (50.00)		
Three	18 (24.32)	6 (22.22)	12 (21.43)	11 (11.58)	47 (18.65)		
Time from surgery to follow-up (years)						26.08	<0.001
<1	15 (20.27)	14 (51.85)	34 (60.71)	49 (51.58)	112 (44.44)		
1–5	59 (79.73)	13 (48.15)	22 (39.29)	46 (48.42)	140 (55.56)		
Family location						0.85	0.843
Urban areas	43 (58.11)	15 (55.56)	28 (50.00)	52 (54.74)	138 (54.76)		
Rural areas	31 (41.89)	12 (44.44)	28 (50.00)	43 (45.26)	114 (45.24)		
Age (year)	3.96 ± 3.10	5.61 ± 4.04	6.48 ± 4.35	6.39 ± 4.66	5.61 ± 4.24	5.92	0.001
Paternal SES index	64.73 ± 20.21	57.45 ± 19.19	64.24 ± 24.56	75.70 ± 53.65	67.98 ± 37.52	2.46	0.063
Maternal SES index	66.59 ± 18.16	56.15 ± 21.40	62.80 ± 26.21	76.75 ± 52.61	68.46 ± 37.16	3.14	0.026

SES, socioeconomic status.

In Cluster 1, the children were the youngest, and the proportion of children who were 1–5 years post-surgery was the highest. In Cluster 2, the proportion of children with cardiac function grade IV was the highest (25.93%), and the maternal SES index was the lowest. In Cluster 3, the proportion of children with biventricular malformation was the highest. In Cluster 4, both the paternal and maternal SES indexes were the highest among the clusters.

### Children's quality of life across different family management styles

Table 3 presents the PedsQL^MT^4.0 scores across the different clusters. Cluster 1 had the highest overall score and Cluster 2 had the lowest overall score. Furthermore, Cluster 2 scored the lowest in all dimensions and was significantly lower than the other three clusters in emotional, social, and school functioning. The differences were statistically significant (*P* < 0.05).

**Table 3:** Scores of each dimension of PedsQL^MT^4.0 in the four clusters.

Family management style	*n*	Physical functioning	Emotional functioning	Social functioning	School functioning	Total
Cluster 1	74	61.28 ± 21.62	70.88 ± 15.78	83.85 ± 15.14	79.73 ± 18.37	73.93 ± 12.71
Cluster 2	27	52.43 ± 25.93	58.33 ± 18.40	64.44 ± 22.93	60.93 ± 24.34	59.03 ± 18.70
Cluster 3	56	59.71 ± 22.24	68.75 ± 17.97	79.20 ± 18.46	71.34 ± 22.49	69.75 ± 17.29
Cluster 4	95	62.37 ± 22.75	65.95 ± 19.87	78.26 ± 19.84	73.58 ± 20.18	70.04 ± 16.90
Total	252	60.39 ± 22.72	67.20 ± 18.43	78.63 ± 19.31	73.53 ± 21.27	69.94 ± 16.53
*F*		1.41	3.44	7.18	5.77	5.66
*P*		0.242	0.017	<0.001	0.001	0.001

### Unplanned readmissions

Table 4 presents the unplanned readmission rates of children across the four clusters. The results of this study showed that the children's quality of life in families in Cluster 1 was the best, while that in Cluster 2 was the worst (*P* = 0.001).

**Table 4:** Comparison of unplanned readmissions of the children in the four clusters.

Family management style	*n*	Number of unplanned readmissions (n)			Unplanned readmission rate (%)	Reasons for unplanned readmission (cases)		
		0	1	≥2		Pulmonary infection	Pleural effusion	Arrhythmia, brain abscess, IE, MOF, and coagulation dysfunction were 1 case each
Cluster 1	74	71	2	1	4.05	3		
Cluster 2	27	22	4	1	18.52	5		
Cluster 3	56	54	2	0	3.57	2		
Cluster 4	95	74	16	5	22.11	13	3	5
Total	252	221	24	7	12.30	23	3	5
*X ^2^*	18.15							
*P*	0.001							

IE, infective endocarditis; MOF, multiple organ failure.

In addition, there were five unplanned readmissions (18.52%) in Cluster 2 due to pulmonary infection, and one child (3.70%) was readmitted twice, Furthermore, parents with the *Laissez-Faire* style (Cluster 4) had the highest rate of unplanned readmissions. Among the 21 unplanned readmissions in Cluster 4, 13 (61.90%) had pulmonary infection, 3 (14.29%) had pleural effusion, and 5 (23.81%) experienced serious complications such as arrhythmia, infectious endocarditis, and multiple organ failure.

### Comparison of family management level between children with CHD and those with other chronic diseases

Compared with a domestic multi-center study on children with other chronic diseases (urinary system, endocrine system, rheumatic, and genetic diseases) (10) (Table 5), the parents of children with CCHD reported similar levels of difficulty in condition management (*P* = 0.473). However, they spent less effort on condition management and demonstrated significantly weaker mutual support (*P* < 0.001).

**Table 5:** Mean scores of the six FaMM subscales in different children (mean ± SD).

FaMM	CHD children who had undergone palliative surgery (*n* = 252)	Children with other chronic diseases (*n* = 399)	*t*	*P*
CDL	17.28 ± 3.84	16.17 ± 5.20	3.12	0.002
CMA	42.27 ± 5.66	41.62 ± 7.44	1.26	0.208
PM	27.90 ± 8.50	33.03 ± 6.66	−8.13	<0.001
VCI	32.54 ± 6.15	31.02 ± 5.88	3.16	0.002
FLD	41.69 ± 10.10	41.07 ± 11.68	0.72	0.473
CME	13.18 ± 3.22	14.63 ± 3.85	−5.18	<0.001

FaMM, Family Management Measure; SD, standard deviation; CDL, Children's Daily Life subscale; CMA, Condition Management Ability subscale; PM, Parental Mutuality subscale; VCI, View of Condition Impact subscale; FLD, Family Life Difficulty subscale; CME, Condition Management Effort subscale.

## Discussion

This study conducted a questionnaire-based investigation of FMS following palliative surgery in children with CCHD. Through cluster analysis, we identified four distinct FMS subtypes. The results revealed that Cluster 1 exhibited the most favorable quality of life, whereas Cluster 2 had the worst. Notably, Clusters 2 and 4 demonstrated significantly higher rates of unplanned readmissions compared to Clusters 1 and 3. Multivariate analysis revealed several potential determinants influencing FMS patterns, including children's age, cardiac function classification, diagnosis, time from surgery to follow-up, and maternal SES index.

FMS captures the nuances of how parents manage the care of their children, offering medical staff insights into how these care plans are woven into the fabric of daily family life. It also sheds light on parents’ perceptions of their children, their own management abilities, and overall family dynamics. Recognizing the variations in FMS among families of children with CCHD post-palliative surgery is crucial for facilitating effective communication and delivering tailored support and interventions.

Among the four clusters examined, FLD exhibited the highest variability (Eta^2^ = 0.59), indicating significant differences in how parents care for children with CCHD. It is widely acknowledged that complex congenital heart palliative surgeries carry a more intricate prognosis compared to straightforward corrective surgeries, along with a higher risk of severe complications, such as renal failure, hemothorax, thrombosis, cerebrovascular accident, multiple organ failure, and the need for additional reoperations. Furthermore, frequent clinical interventions and repeated hospitalizations add to the already substantial burden on these families. Over time, as families gain experience in long-term caregiving, many families have progressively enhanced their ability to navigate these challenges. Thus, the proportion of postoperative time between 1 and 5 years in the Active and Collaborative style cluster (Cluster 1) was the highest (59 cases, 79.73%) among the four clusters.

CDL and PM were two significant positive dimensions in determining the classification of the clusters, highlighting that parents held markedly different perspectives on what constitutes a “normal” daily life for their children. Moreover, the level of mutual support between parents in caring for their children with CCHD varied significantly across the four clusters. PM was found to be positively correlated with CDL and CMA (9). This suggested that promoting effective communication and support between parents could positively influence both the child's daily life and the family's ability to manage the condition. Furthermore, enhancing CMA and reducing the level of FLD could also help reduce CME.

In Chaotic and Nervous families (Cluster 2), the SES index of parents was the lowest among the four family types, and the proportion of children with cardiac function grade IV was the highest (seven cases, 25.93%). Children in these families often received fragmented and passive care, with poor care coordination and limited family participation (14). These families frequently faced insurmountable challenges, which eroded the parents’ confidence in managing their children's conditions. Therefore, clinical staff should prioritize support for these families by understanding the difficulties they encounter in home care, regularly tracking and monitoring their disease management progress, and providing timely support and help. For families with a low SES index, clinical staff can facilitate connections to available community health and/or financial support. Currently, our heart center has expanded its network of regional medical centers to better serve economically disadvantaged families of patients with CHD in remote areas, ensuring they receive the care and support they need.

In addition, compared to a cross-sectional study (15.9%) (15), the children in Clusters 2 and 4 had a significantly higher unplanned readmission rate. This underscores the critical impact of insufficient attention to postoperative care, including infection prevention, feeding, and follow-up, which can lead to various adverse outcomes. Therefore, it is important for healthcare workers to provide adequate disease health education and regular assessment of children in the families with Chaotic and Nervous (Cluster 2) and *Laissez-Faire* styles (Cluster 4).

Last but not least, we compared the families of children with CCHD to those of children with other chronic diseases. Table 5 shows that families of children with CCHD were more concerned about their children's prognosis compared to families of children with other chronic illnesses. Moreover, the level of mutual support among these families was far weaker than in families of children with other chronic illnesses. Therefore, our findings highlight the need for a centered, multi-team intervention approach to enhance mutual support among families of children with CCHD.

### Limitations

First, the FaMM data were collected from only one parent and the results were based on their subjective perceptions. This may cause bias to some extent, as some findings might reflect the perspectives of the adult completing the survey rather than the cardiac problems. Second, this study only conducted a cross-sectional survey of the status of FMS without long-term follow-up. A longitudinal study will be needed to assess the stability of FMS and its relation to readmission and control for confounders, including the severity of disease and success of the surgery. In the future, we will implement an evaluation study to examine different interventions, including the standard of care, and long-term outcomes of children with CCHD.

## Conclusion

The novel contribution of the study lies in the different FMSs after palliative surgery in families of children with CCHD. These varying styles reflected diverse care priorities and characteristics across different families. An effective FMS has been shown to positively influence the outcomes of children. Therefore, health managers should implement family-centered interventions and guide families in adopting an effective FMS to improve the outcomes of children with CCHD.

## Data Availability

The original contributions presented in the study are included in the article/Supplementary Material, further inquiries can be directed to the corresponding authors.
